# Dent Disease Type 2 as a Cause of Focal Segmental Glomerulosclerosis in a 6-Year-Old Boy: A Case Report

**DOI:** 10.3389/fped.2020.583230

**Published:** 2020-10-28

**Authors:** Martin Bezdíčka, Jan Langer, Jaromír Háček, Jakub Zieg

**Affiliations:** ^1^Department of Pediatrics, Second Faculty of Medicine, Charles University in Prague and Motol University Hospital, Prague, Czechia; ^2^Department of Pediatrics and Adolescent Medicine, First Faculty of Medicine, Charles University and General University Hospital in Prague, Prague, Czechia; ^3^Department of Pathology and Molecular Medicine, Second Faculty of Medicine, Charles University in Prague and Motol University Hospital, Prague, Czechia

**Keywords:** case report, Dent disease, focal segmental glomerulo sclerosis, nephrotic syndrome, OCRL gene

## Abstract

Dent disease is an X-linked recessive renal tubular disorder characterized by proximal tubule dysfunction. Typical features include low molecular weight proteinuria, hypercalciuria, nephrocalcinosis, nephrolithiasis, rickets, and chronic renal failure. We present a case of a 6-year-old boy with nephrotic proteinuria without hypoalbuminemia or edema. His renal biopsy revealed focal segmental glomerulosclerosis (FSGS), some of the glomeruli were globally sclerotic. Hypercalciuria was present intermittently and urine protein electrophoresis showed low molecular weight protein fraction of 50%. The next generation sequencing identified pathogenic variant in *OCRL* gene causing Dent disease type 2. We report an uncommon histologic finding of FSGS in Dent disease type 2 and highlight the importance of protein content examination and genetic analysis for the proper diagnosis in these complicated cases.

## Introduction

Dent disease is an X-linked recessive renal tubular disorder, which primarily affects males. Females are mostly heterozygous carriers of one affected X chromosome, without a disease phenotype. The probability of affected males in the next generation is 50%, because men only have one X chromosome, inherited from their mother. Dent disease is characterized by low molecular weight proteinuria, rickets, hypercalciuria, and nephrocalcinosis. This disorder frequently leads to the formation of stones in the urinary tract and progressive chronic kidney disease (CKD) with some features of Fanconi syndrome (1). The *OCRL* variant p.Arg318Cys has been described in association with less common Dent disease type 2 and segregates in an X-linked recessive manner (cytogenetic band Xq26.1) with the disease according to previous literature (2). Pathogenic variants in the *OCRL* gene, which encodes specific lipid phosphatase, may affect the regulation of membrane trafficking, actin dynamics or the modulation of phospholipids, leading to the clinically different form than more frequent Dent disease type 1, which is associated with mutated *CLCN5* gene and has usually more serious phenotype) (3). Interestingly, variants in the *OCRL* gene were found to cause oculo-cerebro-renal syndrome (Lowe syndrome), a multisystem disorder characterized by Fanconi syndrome, progressive CKD, congenital cataract, and cognitive difficulties. Patients with Dent disease type 2 may suffer from a mild form of psychomotor delay, hypotonia, and cataract, thus representing an intermediate phenotype between Dent disease and Lowe syndrome (4). Low sodium diet and thiazide diuretics are the mainstay of therapy. At the same time, hypovolemia and nephrolithiasis should be prevented. Other metabolic disturbances (e.g., acidosis, hypokalemia, rickets) are treated by supplementation. Some patients require conservative and surgical management of lithiasis. During the course of the disease, CKD complications evolve and need to be managed accordingly. The majority of patients with Dent disease reach end stage kidney disease between the ages of 30 and 50 years (1). Interestingly, one study showed protective effect of high citrate diet on renal insufficiency in ClC-5 knockout mouse model of Dent disease (5). However, these results have not been replicated in humans yet. Here, we report a case of Dent disease type 2 associated with an unusual histologic finding of focal segmental glomerulosclerosis (FSGS).

## Case Report

**Figure 1:**
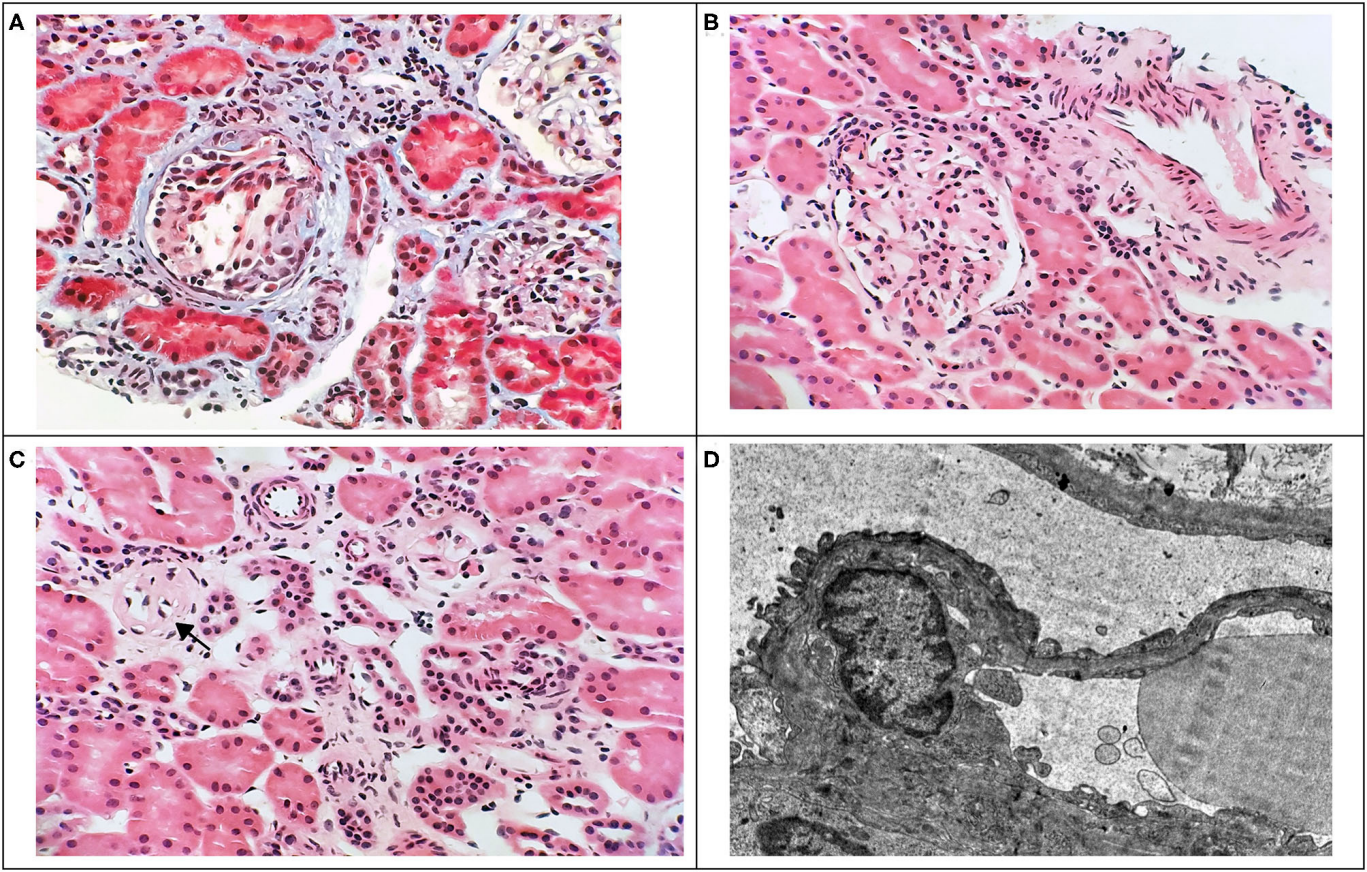
Renal biopsy findings. Light microscopy of renal tissue showing glomerular tuft collapse with podocyte hypertrophy **(A)**, focal segmental mild mesangial hypercellularity **(B)**, and global glomerulosclerosis **(C)** with mild focal interstitial fibrosis and mild lymphocytic interstitial infiltrate **(A,C)**. (**A**: Masson's trichrome, x400; **B,C**: HE, x400). Electron microscopy image of renal tissue **(D)** showing foot processes effacement (x8000).

**Figure 2:**
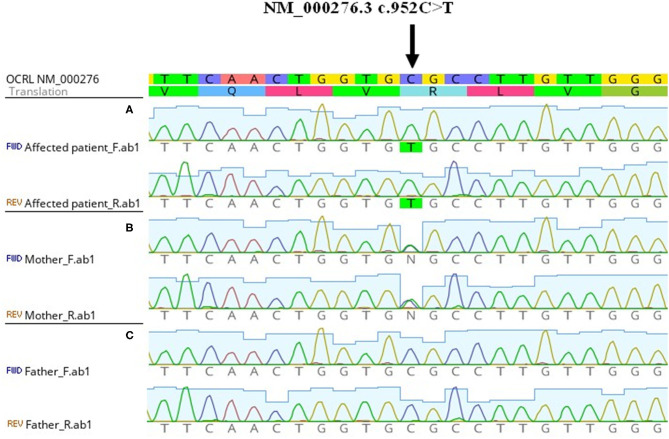
Genetic finding of *OCRL* pathogenic variant. From above: nucleotide reference sequence, amino acid reference sequence, electropherogram from Sanger sequencing of aligned forward and reverse sequences. The picture was obtained from Geneious Prime software. **(A)** Hemizygous NM_000276.3 c.952C>T in affected patient. **(B)** Heterozygous NM_000276.3 c.952C>T in the mother without a disease phenotype. **(C)** Negative sequencing results of the healthy father.

A 6-year-old boy was referred with the accidental finding of nephrotic range proteinuria when examined for nocturnal enuresis to the tertiary center. On admission, he was normotensive, without peripheral edemas, and physical examination was normal. The family history revealed that his maternal uncle was being followed-up for proteinuria of unknown etiology. Our patient's past medical history was unremarkable; he did not take any medication. His mild motor development delay required temporary reflex locomotion therapy. His height and weight were in the 10th and 25th percentiles for age, respectively. The laboratory findings were as follows: white blood cell count 11.1 × 10^9^/L, hemoglobin 134 g/dL, and platelets 272 × 10^9^/L. Serum creatinine was 49 μmol/L, potassium 4.7 mmol/L, chloride 100 mmol/L, bicarbonate 24.9 mmol/L, cholesterol 5.6 mmol/L, triglycerides 1.03 mmol/L, albumin 49.8 g/L, total protein 75 g/L. His urinalysis revealed 2+ protein, a protein to creatinine ratio of 315 mg/mmol, microalbumin to creatinine ratio of 58 mg/mmol, calcium to creatinine ratio of 0.6 mmol/mmol, urine examination showed 13 erythrocytes/μL and no leukocytes. Infectious disease screening was conducted including EBV, CMV, gonorrhea and HIV serology, with negative results. Immunology tests showed mild hypogammaglobuminemia of 5.19 g/L, C3 and C4 levels were within normal range: 0.73 and 0.22 g/L, respectively. Also, antinuclear antibodies, anti-neutrophil cytoplasmic antibodies and the extractable nuclear antigen panel were all negative. Renal ultrasound showed that both kidneys were of normal size. There was no evidence for renal cysts, kidney stones, nephrolithiasis, or nephrocalcinosis. At this time, the patient was referred to tertiary center for percutaneous diagnostic renal biopsy which showed histologic features consistent with FSGS. Approximately 30% of glomeruli showed mild segmental mesangial hypercellularity. Capillary tuft was globally collapsed in one glomerulus and segmental collapse with adhesion to Bowman's capsule was present in three glomeruli. Two glomeruli were globally sclerotic. Only sparse small foci of interstitial fibrosis were revealed. Electron microscopy showed segmental capillary collapse, podocyte foot processes effacement (~70% of loops) together with podocyte hypertrophy and microvillous change, see Figure 1. The administration of corticosteroids was considered; however, due to the absence of hypoalbuminemia and edemas, primary FSGS was not probable and only ramipril was prescribed. Urine protein electrophoresis was performed and showed 50% of low molecular weight proteinuria. Also urine concentration of renal tubular damage markers (N-acetyl β-D-glucosaminidase 192 nkat/L, alpha 1 microglobulin 279 mg/mL) was significantly increased, while albuminuria was only moderately increased. Hypercalciuria was present intermittently. These examinations increased suspicion for tubulopathy. The DNA sample of the patient was analyzed by next generation sequencing. The libraries were prepared according to the manufacturer's protocol and sequenced with a NextSeq 500/550 High Output Kit v2.5 (150 cycles) on a NextSeq 550 instrument (Illumina, San Diego, CA). The sequenced data were filtered and evaluated using an Ingenuity variant analyser according to current standards of the American College of Medical Genetics and Genomics, as described previously (6). The analysis revealed a known pathogenic variant c.[952C>T];[0], p.[(Arg318Cys)];[0] (rs137853263 in the dbSNP database) in the *OCRL* gene (NM_000276.3, NP_000267.2). This variant has been described in association with Dent disease type 2 and segregates in an X-linked recessive manner (cytogenetic band Xq26.1) with the disease. No other causative variants in known FSGS-associated genes were found. Sanger sequencing of parental DNA samples confirmed the presumed inheritance of the variant. The patient's mother is a healthy carrier of the c.952C>T p.(Arg318Cys) variant, while analysis of the father's DNA was negative (Figure 2).

The patient was prescribed hydrochlorothiazide at the dose of 1 mg/kg/day, ramipril 5 mg daily and phosphate supplementation. He suffered an episode of prerenal acute kidney injury (AKI) due to acute gastroenteritis after one year from the establishment of the diagnosis. Parenteral rehydration lead promptly to complete resolution of AKI. His renal function remained stable, his acid-base status was normal, the proteinuria persisted in nephrotic range, renal ultrasound showed adequate kidney growth without nephrocalcinosis or nephrolithiasis on last observation. The patient has been followed up in good clinical condition for 2 years in total.

## Discussion

Primary FSGS is usually found in patients with nephrotic syndrome (NS) and thought to be caused by a circulating permeability factor, which is responsible for increased leakiness of the glomerular filtration barrier, leading to proteinuria. A number of molecules have been proposed to play this role; unfortunately, despite the efforts of many researchers, this factor has not yet been identified (7). On the other hand, FSGS may be secondary to infections (HIV, parvovirus B19, EBV, CMV), drugs (pamidronate, lithium, sirolimus, interferon, heroin), and causative variants of the genes encoding proteins important for the proper structure and function of the filtration barrier. In addition, various injuries to the kidney (reduced renal mass, systemic hypertension, obesity, sickle cell disease, cyanotic heart disease) may cause FSGS (8). Of interest, our patient was not a typical child with NS, because nephrotic proteinuria was not accompanied by hypoalbuminemia, or by edema. This raised the suspicion of a hereditary etiology of the nephropathy. Moreover, the significant proportion of tubular proteinuria pointed to inherited or acquired proximal tubular dysfunction. It has been shown that there is a significant relationship between the selectivity of proteinuria and tubulointerstitial damage in patients with NS (9).

Initially, nonselective proteinuria was considered to be the main cause of injury to renal tubules in FSGS. Although certain pathogenic mechanisms for tubular damage were proposed, there was no clear evidence for the direct toxicity of the protein to the tubulointerstitium (10). This assumption was further challenged by other researchers who proposed that tubular dysfunction might precede the development of FSGS in some cases. Salt-wasting inherited tubulopathies, Bartter syndrome and Gitelman syndrome, have been linked to the development of FSGS. Furthermore, the association of hereditary distal renal tubular acidosis with FSGS has been reported in the literature. There are also few case reports of FSGS seen in Lowe and Dent disease kidney histology (11–13). To the best of our knowledge, only one patient with Dent disease type 2 and FSGS has been reported previously (14). Stimulation of the renin-angiotensin system and the subsequent production of profibrotic cytokines is considered to be the main mechanism leading to sclerotic changes and progressive damage in the renal tissue of patients with tubulopathies (15). Predominant histologic findings in Dent disease are global glomerulosclerosis and interstitial fibrosis. Various tubulointerstital involvements may be present, including tubular atrophy, dilatation, and interstitial lymphocytic infiltration. Nephrocalcinosis is only detected in a minority of patients and its presence does not influence the decline of the glomerular filtration rate. Immunofluorescence is either negative or nonspecific (13). Our patient had both focal segmental and global sclerotic glomerular lesions with mild focal interstitial fibrosis and lymphocytar infiltration; nephrocalcinosis was not detected.

With time, progression of glomerular scarring in FSGS may lead to global glomerulosclerosis and both focal segmental and global sclerosis may be seen (12). While primary forms of FSGS may respond to immunosuppressants or plasma exchange, maladadaptive forms associated with reduced nephron numbers or abnormal stress to nephrons should be treated with renin angiotensin system inhibitory drugs. Drug-induced or virus-associated FSGS are managed by the discontinuation of medication or the resolution or active treatment of infection. The presence of nephrotic proteinuria with normal serum albumin and the absence of edema suggest an etiology other than primary FSGS (16). When evaluating children with nephrotic proteinuria and a high level of tubular proteins, tubulopathy needs to be considered early in the diagnostic process. Genetic testing should be focused also on genes associated with tubulopathies. The clinical presentation in Dent disease may differ, as cataracts, mental retardation, acidosis, nephrocalcinosis, and kidney stones may be absent in Dent disease type 2 or can occur variably in affected individuals (2). Also, hypercalciuria may be present but is not a constant finding, as in our patient (11). Importantly, low molecular weight proteinuria should direct the clinician toward the possibility of the phenotypic spectrum of Dent disease and Lowe syndrome. The heterogeneity of symptoms among Dent disease type 2 patients with a confirmed pathogenic variant in *OCRL* can be caused by another factor that could compensate for the function of OCRL phosphatase. The p.(Arg318Cys) variant disrupts the catalytic phosphatase domain and probably reduces protein stability. This protein instability can lead to the elevation of phosphatidylinositol-4,5-bisphosphate and disruption of the actin cytoskeleton, which can influence many trafficking systems and cellular processes (2, 3). The diverse types of disruption may have different consequences, like nonspecific FSGS developed by Dent disease type 2. A proper diagnosis enabled our patient to avoid unnecessary exposure to immunosuppressive treatment in FSGS patients, and symptomatic therapy could be started.

## Conclusion

In conclusion, the genetic diagnosis of X-linked recessive Dent disease type 2 provided information about the expected course and outcome of the disease for the patient and his family. This case report highlights the importance of a complex view of FSGS as a heterogeneous entity. Clinicians need to investigate the urine protein content, as different proteins present may reflect the site of renal injury and help to make a proper diagnosis. The presence of only mildly elevated albuminuria in a child with nephrotic proteinuria should point to tubular disease. Also in patients with nephrotic proteinuria and high tubular content immunosuppressive therapy should be avoided before kidney biopsy performance. Finally, a positive family history should alert the physician to the high possibility of the genetic predisposition of the disease. Genetic analysis is essential in establishing the diagnosis in such complex cases.

## Data Availability Statement

The original contributions presented in the study are included in the article/supplementary material, further inquiries can be directed to the corresponding author/s.

## Ethics Statement

All procedures performed in studies involving human participants were in accordance with the ethical standards of the institutional research committee (Ethics Committee for Multi-Centric Clinical Trials of the Motol University Hospital and 2nd Faculty of Medicine, Charles University in Prague) and with the 1964 Helsinki declaration and its later amendments or comparable ethical standards. Informed consent was obtained from all individual participants included in the study. Written informed consent from the parents of the patient was obtained for the publication of any potentially identifiable images or data included in this article.

## Author Contributions

MB and JZ performed the genetic analysis and wrote the manuscript. JL and JH helped with clinical data and critically reviewed the manuscript. All authors contributed to the article and approved the submitted version.

## Conflict of Interest

The authors declare that the research was conducted in the absence of any commercial or financial relationships that could be construed as a potential conflict of interest.

## Abbreviations

AKI: acute kidney injury
CKD: chronic kidney disease
FSGS: focal segmental glomerulosclerosis
NS: nephrotic syndrome.
